# Identification of Putative Steroid Receptor Antagonists in Bottled Water: Combining Bioassays and High-Resolution Mass Spectrometry

**DOI:** 10.1371/journal.pone.0072472

**Published:** 2013-08-28

**Authors:** Martin Wagner, Michael P. Schlüsener, Thomas A. Ternes, Jörg Oehlmann

**Affiliations:** 1 Department Aquatic Ecotoxicology, Faculty of Biological Sciences, Goethe University Frankfurt, Frankfurt am Main, Germany; 2 Federal Institute of Hydrology (BfG), Koblenz, Germany; University of Geneva, Switzerland

## Abstract

Endocrine disrupting chemicals (EDCs) are man-made compounds interfering with hormone signaling and thereby adversely affecting human health. Recent reports provide evidence for the presence of EDCs in commercially available bottled water, including steroid receptor agonists and antagonists. However, since these findings are based on biological data the causative chemicals remain unidentified and, therefore, inaccessible for toxicological evaluation. Thus, the aim of this study is to assess the antiestrogenic and antiandrogenic activity of bottled water and to identify the causative steroid receptor antagonists. We evaluated the antiestrogenic and antiandrogenic activity of 18 bottled water products in reporter gene assays for human estrogen receptor alpha and androgen receptor. Using nontarget high-resolution mass spectrometry (LTQ-Orbitrap Velos), we acquired corresponding analytical data. We combined the biological and chemical information to determine the exact mass of the tentative steroid receptor antagonist. Further MS^n^ experiments elucidated the molecule’s structure and enabled its identification. We detected significant antiestrogenicity in 13 of 18 products. 16 samples were antiandrogenic inhibiting the androgen receptor by up to 90%. Nontarget chemical analysis revealed that out of 24520 candidates present in bottled water one was consistently correlated with the antagonistic activity. By combining experimental and *in silico* MS^n^ data we identified this compound as di(2-ethylhexyl) fumarate (DEHF). We confirmed the identity and biological activity of DEHF and additional isomers of dioctyl fumarate and maleate using authentic standards. Since DEHF is antiestrogenic but not antiandrogenic we conclude that additional, yet unidentified EDCs must contribute to the antagonistic effect of bottled water. Applying a novel approach to combine biological and chemical analysis this is the first study to identify so far unknown EDCs in bottled water. Notably, dioctyl fumarates and maleates have been overlooked by science and regulation to date. This illustrates the need to identify novel toxicologically relevant compounds to establish a more holistic picture of the human exposome.

## Introduction

By interfering with the organism’s complex hormone signaling endocrine disrupting chemicals (EDCs) might adversely affect development and reproduction [1], [2]. Moreover, recent research suggests an implication of EDCs in cancer, cardiovascular, and metabolic disorders [3], [4], [5]. While research generates an ever-growing list of potential EDCs, few compounds, namely Bisphenol A (BPA) and phthalates, attract particular scientific attention and public controversy. Used in a vast variety of consumer products, these chemicals are ubiquitously detected in the environment as well as in human samples [6], [7], [8]. With numerous studies documenting adverse effects [9], [10], public health concerns have led to a voluntary or regulatory removal of BPA and phthalates in some products (e.g., baby bottles, toys) and countries.

However, given the multitude of chemicals in use, these measures might not resolve the problem. This is illustrated by a recent study suggesting that plastic products marketed as BPA free release significant amounts of estrogenic activity [11]. The authors employed a sensitive *in vitro* bioassay to characterize the total estrogenic burden leaching from plastics, including potential mixture effects and unidentified EDCs. Using a similar approach, a series of studies reported a widespread estrogenic contamination of commercially available bottled water [12], [13], [14], [15], [16], [17]. Another study adds to the picture by presenting new findings on androgenic, antiandrogenic, progestagenic, and glucocorticoid-like activity in bottled water [16]. Attempts to explain the observed effects by targeted chemical analysis remained unsuccessful [18] and it has soon become clear that ‘traditional’ EDCs are not responsible for the endocrine activity in bottled water. Since the causative chemical entity remains so far unidentified [19], the findings are not easy to interpret in a toxicological context and, consequently, prone to criticism [20].

Here, we combine biological and chemical analysis to identify putative steroid receptor antagonists in bottled water. Most of the products were potently antiestrogenic and antiandrogenic in the bioassays. Nontarget high-resolution mass spectrometry pointed towards maleate and fumarate isomers as promising candidates and subsequently enabled the identification of di(2-ethylhexyl) fumarate. Because its concentration is too low to explain the observed activity, other compounds must contribute. However, further maleate/fumarate isomers are not only biologically active but structurally highly similar to phthalates. Hence, we speculate these compounds might represent a novel, so far overlooked group of EDCs.

## Methods

### Reagents

All reagents used for sample preparation and bioassays have been previously reported [17], [21]. Reagents for chemical analysis were the purest grade available. 2-Butenedioic acid (2Z)-, 1,4-bis(2-ethylhexyl) ester (di(2-ethylhexyl) maleate, DEHM, CAS 142-16-5), 2-Butenedioic acid (2E)-, 1,4-bis(2-ethylhexyl) ester (di(2-ethylhexyl) fumarate, DEHF, CAS 141-02-6), 2-Butenedioic acid (2Z)-, 1,4-dioctyl ester (dioctyl maleate, DOM, CAS 2915-53-9) were purchased from Sigma-Aldrich (Steinheim, Germany). 2-Butenedioic acid (2E)-, 1,4-dioctyl ester (dioctyl fumarate, DOF, CAS 2997-85-5) was purchased from Angene Intl. (Hong Kong, PR China).

### Samples and Sample Preparation

Samples and sample extraction procedures have been described in detail previously [17]. In brief, 18 different bottled waters (coded as samples 1–18) produced by 13 different companies in France, Germany, and Italy were purchased in local supermarkets.

To optimize the extraction of steroid receptor antagonists, we applied the same strategy as previously described [17]. First, one brand of bottled water (sample 18) was extracted using six different solid phase extraction (SPE) sorbents. Tap water extracted identically served as procedural blank. In addition, empty SPE cartridges were extracted to control for a potential contamination of the materials. All extracts were analyzed for antiestrogenic activity in the Yeast Antiestrogen Screen (see below). The extracts of empty SPE cartridges and tap water did not induce significant antiestrogenicity (Figure S1, S2). This indicates that neither materials nor procedure cause a contamination of the samples. An SPE method employing Isolute ENV+ cartridges (200 mg, Biotage, Uppsala, Sweden) successfully extracted antiestrogenic activity from bottled water (Figure S2).

Accordingly, this method was applied to the full sample set: 1.5 L of each sample was degassed and extracted with ENV+ cartridges. Samples were eluted with 4 mL methanol. 100 µL DMSO was added as keeper. Methanol was removed under a gentle stream of nitrogen yielding a final extract of 100 µL DMSO (concentration factor 15,000). Identically treated tap water served as procedural blank in all extractions. In addition, all used solvents were concentrated like the extracts and analyzed for potential contamination. The extraction was independently repeated three times. All extracts were stored in glass vials with PTFE cap at −20°C prior to further analysis.

### Bioassay

The antiestrogenic and antiandrogenic activity of bottled water extracts was evaluated in reporter gene assays for human estrogen receptor alpha (Yeast Antiestrogen Screen, YAES) and human androgen receptor (Yeast Antiandrogen Screen, YAAS). To detect antagonists, Yeast Estrogen Screen [22] and Yeast Androgen Screen [23] were modified to analyze samples in the presence of the endogenous ligand of each receptor (17β-estradiol, testosterone). Receptor antagonists present in the sample cause a displacement of the endogenous ligand resulting in a decreased reporter gene signal.

The general assay procedures have been described previously [21], [24]. Briefly, SPE extracts were diluted 480 fold in assay medium and coincubated with 30 pM 17β-estradiol (YAES) or 2.5 nM testosterone (YAAS) dissolved in ethanol. Negative controls, solvent controls (ethanol and DMSO) with and without endogenous ligand, and positive controls (ligand coincubated with a known receptor antagonist, YAES: 80 µM hydroxytamoxifen, YAAS: 50 µM flutamide) were included in each experiment. The maximum solvent concentration was 0.4% v/v in all cases. Samples were analyzed in eight replicates, controls in 8–48 replicates. After 24 hours incubation at 30°C turbidity was read to assess cytotoxicity and β-galactosidase activity was determined [12]. Samples from three extractions were tested in three independent YAES and YAAS experiments each.

To determine the relative inhibition of estrogen and androgen receptor, the samples’ corrected absorbance (OD) determined in each experiment was normalized to the adequate controls containing the ligand 17β-estradiol or testosterone (OD_C+E2/T_, 0% inhibition) and without ligand (OD_C-E2/T_, 100% inhibition) as follows: relative inhibition = 100– ((OD_sample_-OD_C-E2/T_)/(OD_C+E2/T_-OD_C-E2/T_) ×100). Inhibition data is reported as means of three SPE extracts per sample analyzed in three experiments (with eight replicates each) resulting in a sample size of 63–72. The reported antagonistic activity corresponds to a sample volume equivalent to 3.75 mL bottled water.

### High-resolution Mass Spectrometry (LTQ-Orbitrap Velos)

For the chemical analysis 110 µL methanol was added to 40 µL SPE extract. Samples from two independent extractions were analyzed in two LC-ESI-LTQ-Orbitrap experiments. Chromatographic conditions were the same as for quantification by LC-tandem MS. The ESI source parameters for positive ionization were set as follows: capillary temperature: 350°C; capillary voltage 3.5 kV; heater temperature 400°C; sheath gas flow rate 40 arbitrary units (AU); aux gas flow rate 15 AU; S-lens RF level 67%. Data dependent acquisition was used to conduct MS^2^ and MS^3^ spectra as follows: a full scan (120–1200 m/z; positive mode) was performed followed by MS^2^ and MS^3^ scans for the two most intense ions with an intensity of >10,000 counts per second (cps) and >1,000 cps, respectively. Collision induced dissociation (CID) with a normalized collision energy of 35% was used for fragmentation with an activation time of 10 ms. In addition, dynamic exclusion was applied (exclusion of masses for which three MS^n^ experiments have been performed; exclusion duration: 30 s) enabling also MS^n^ experiments for less abundant ions (e.g., during co-elution of different substances). Analysis using negative ionization was performed at similar conditions (details not shown here).

### Chromatographic Conditions for Orbitrap and LC-tandem MS Studies

Separations were performed using a Luna C18(2) column (2 mm i.d., length 150 mm, particle size 3 µm) and a SecurityGuard (both Phenomenex, Torrance, CA, USA) at 30±2°C. The flow rate was 0.2 mL/min. The HPLC gradient was established by mixing two mobile phases. Phase A: MilliQ water and phase B: methanol. Chromatographic separation was achieved with the following gradient: 0–1 min: 0% B; 1–19 min: 0→100% B; 19–29 min: 100% B; 29–29.1 min: 100→0% B; 29.1–35 min: 0% B. 10 µL of each sample was injected.

### Combining Nontarget Chemical Analysis and Bioassay Data

We used the open-source software MZmine 2.2 to process our analytical data [25]. For each sample, a peak list was generated applying the chromatogram builder and peak deconvolution algorithms. For each LTQ-Orbitrap experiment, deisotoped peak lists were aligned to match corresponding peaks in the multiple samples. Finally, the peak finder algorithm was used to secondarily identify peaks missed during peak detection.

Subsequently, these peak lists were filtered for peaks present in at least twelve samples. To identify peaks coinciding with the biological activity, we correlated the areas of each peak (corresponding to its concentration) with the antiestrogenic and antiandrogenic activity of the corresponding samples. From that, we selected the peaks that correlated significantly with the inhibition in the YAES and/or YAAS (p<0.05).

To further narrow down the number of candidates we combined the peak lists of the two independent Orbitrap experiments and filtered for peaks that were consistently detected in both analytical runs with a maximum m/z difference of 10 ppm. Peaks with contradicting r-values in both experiments were excluded as implausible. For the remaining candidates, we checked the extracted ion chromatograms (XIC) and excluded those, which were misinterpreted as peak by MZmine. For the final candidates, we reanalyzed the exact masses (m/z), retention times, and peak areas in the raw data (Xcalibur 2.1, Thermo Fisher Scientific Inc., San Jose, USA). Scatter plots of the z-transformed peak areas and the antiestrogenic and antiandrogenic activity were used to assess the plausibility of correlation. This procedure results in one remaining m/z candidate.

All statistical analysis was performed using Graphpad Prism (5.03, GraphPad Software, Inc., San Diego, USA) and STATISTICA (8.0, StatSoft, Inc., Tulsa, USA). Nonparametric Kruskal–Wallis tests (with Dunn’s multiple comparison test) were applied to compare bioassay data. A p value of <0.05 was regarded as significant.

### Structural Elucidation and Identification

To gain information on the final candidate’s molecular structure we conducted MS^n^ experiments: Samples with high concentrations of the target compound (samples 13, 18) were analyzed using the same experimental conditions as described above. The exact mass of the final candidate (mean of all experiments and samples) was searched in the ChemSpider database. Chemical structures including possible adducts that matched the exact mass within a range of 10 ppm were subjected to *in silico* fragmentation using Mass Frontier 6.0 (HighChem, Ltd., Bratislava, Slovakia). The experimental and predicted fragmentation spectra were compared.

### Confirmation

Chemicals with best matching fragmentation and plausible structure (DEHM, DOM, DEHF, DOF) were purchased as authentic standards. Their antiestrogenic and antiandrogenic activity was evaluated in the bioassays (three experiments) as described above in concentrations ranging from 300 nM to 1 mM (in ethanol).

The HPLC system consisted of a G1367C autosampler, a G1312B binary HPLC pump, a G1379B degasser (all Agilent 1200 SL Series, Waldbronn, Germany) and a MistraSwitch column oven (MayLab Analytical Instruments, Wien, Austria). The detection was performed on a API 4000 Qtrap mass spectrometer (with turboionspray ionization, Applied Biosystems, Foster City, CA, USA).

The tandem MS was operated in positive ion mode using nitrogen as collision gas and multiple reaction monitoring (MRM) for quantification. Parameters adjusted were collision gas (CAD), 6 mTorr; curtain gas (CUR), 20 psi; ion source gas 1 (GS1), 30 psi and ion source gas 2 (GS2), 40 psi; source temperature (TEM), 500°C; entrance potential (EP), 10 V. The ionspray voltage (IS) was adjusted to 5.5 kV and the interface heater (ihe) set on. Two MRM transitions for each substance were monitored for identification and quantification of the analytes. Parameters such as declustering potential, collision energy, and cell exit potential were optimized in the auto-tuning routine of the Analyst 1.4.2 software. Table S5 gives an overview of all MS parameters. For chromatographic conditions see above.

## Results

### Optimizing Solid Phase Extraction

Using a similar approach as described previously [17] we evaluated different SPE methods to isolate steroid receptor antagonists from bottled water. Quality control experiments indicate that neither the solvents nor the cartridges used for extraction lead to a contamination of extracts with antiestrogens (Figure S1). Likewise, tap water extracted as procedural blank according to each method did not induce significant antiestrogenic activity in the YAES (Figure S2). When comparing the extraction efficiency, one SPE method (employing Isolute ENV+ cartridges) yielded an extract that was significantly antiestrogenic. This indicates that only the ENV+ sorbent (hydroxylated styrene-divinylbenzene) was able to extract antiestrogens from bottled water effectively (Figure S2).

### Steroid Receptor Antagonists in Bottled Water

**Figure 1:**
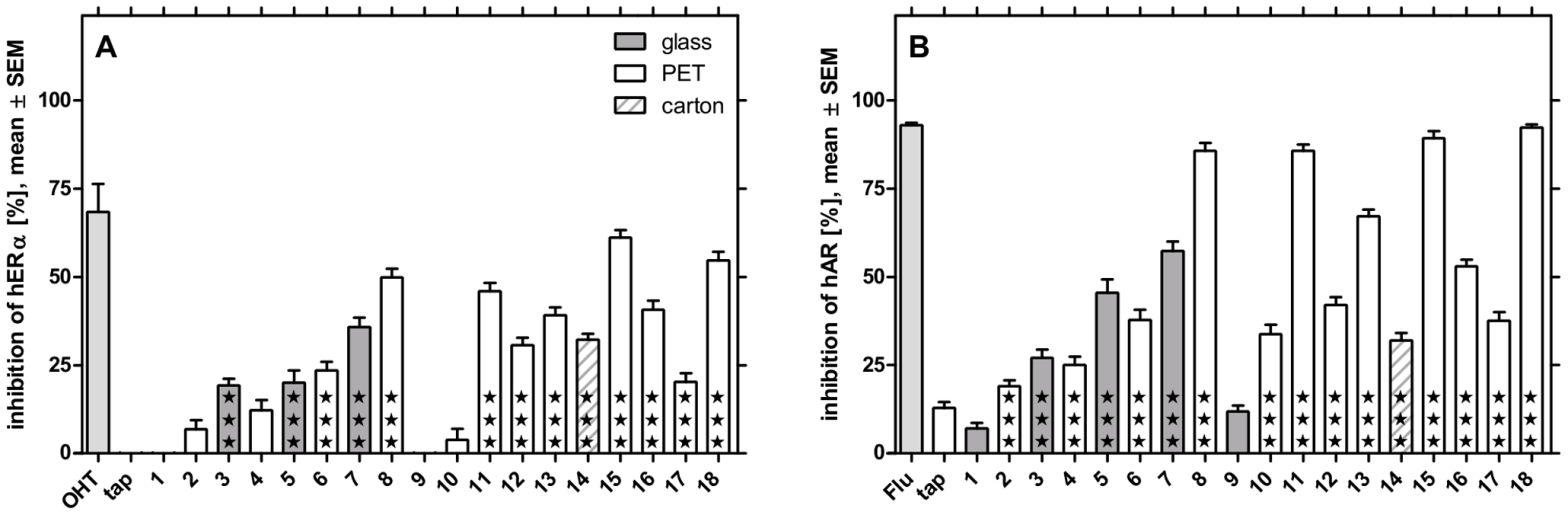
Antiestrogenic (A) and antiandrogenic activity (B) of 18 bottled waters. 13 waters significantly inhibit estrogen receptor alpha, 16 samples antagonize androgen receptor (★★★p<0.0001, compared to controls with endogenous ligand). The activity was normalized to controls containing 17β-estradiol or testosterone (0% inhibition) and such without (100% inhibition). The results represent three extracts per sample tested in three experiments with eight replicates each.

Extracted with the optimized method, the majority of bottled water products significantly inhibited human estrogen as well as androgen receptor. In the YAES 13 products were antiestrogenic (Figure 1 A) with an inhibition of 19.2 (±1.97) to 61.1 (±2.09)%. We detected significant antiandrogenic activity in 16 samples in the YAAS (Figure 1 B). Here, antagonistic activity ranged from 19.0 (±1.66) to 92.3 (±0.88)%. The samples’ potential to antagonize estrogen and androgen receptor is significantly correlated (p<0.001, r = 0.937, Figure S3). Tap water extracted as blank did not induce any significant activity documenting that the procedure does not lead to a contamination with antiestrogens or antiandrogens.

### Combining Nontarget Chemical Analysis and Bioassay Data

We analyzed two distinct SPE extracts per sample in two independent Orbitrap experiments using positive and negative ionization. Following our data analysis strategy, initial data processing with MZmine generated two lists with 15593 and 24520 peaks detected in the samples in the positive mode (Table S1). These lists were restricted to peaks present in at least twelve samples resulting in 12,466 and 18,685 peaks, respectively. We then correlated the areas of these peaks with the antiestrogenic and antiandrogenic activity and identified 938 and 1066 candidates that were significantly correlated with the antagonistic activity in the YAES and/or the YAAS (p<0.05). Out of these, 67 candidates were detected in both Orbitrap experiments (Table S2). To further narrow down this list we excluded candidates with inconsistent correlations, i.e., correlation coefficients are positive in one and negative in the other experiment. We assessed the extracted ion chromatograms (XIC) of the remaining 43 candidates and selected only those with plausible chromatograms in both Orbitrap experiments. Consequently, 40 peaks, which MZmine generated from noise, were discarded.

**Figure 2:**
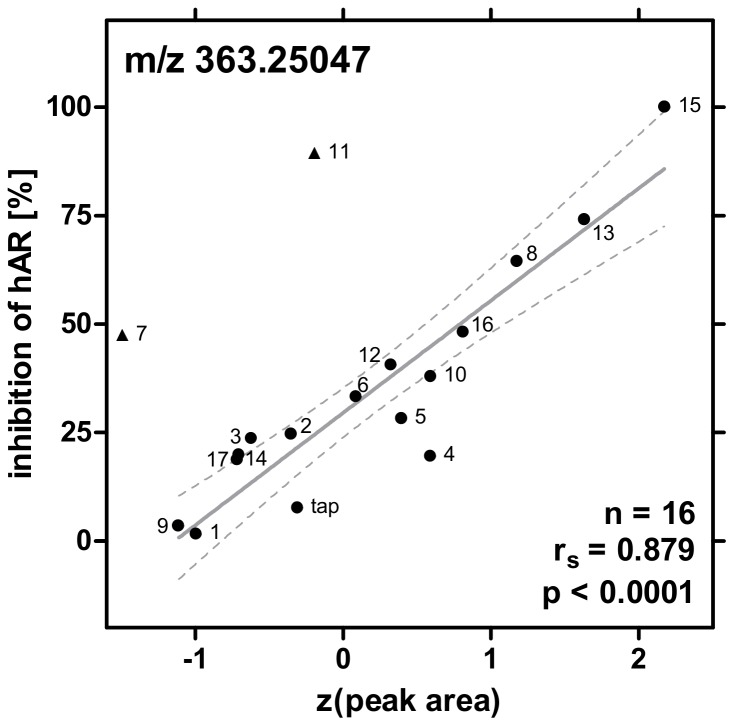
Correlation of the peak areas (Z-transform) of the final candidate (exact mass 363.25047) with the antiandrogenicity. Data from Orbitrap experiment 1 is shown here exemplarily. Triangles indicate outliers, the linear regression (with 95% confidence bands) is shown in grey.

From the raw data we manually reanalyzed the exact masses (m/z), retention times, and peak areas of the three remaining candidates (m/z 229.14103, 352.09008, and 363.25047). Correlation of the recalculated peak areas with the biological activity indicated that two candidates (229.14 and 325.09) were only loosely and in some cases not significantly correlated in the individual experiments (Figure S4). In addition, these candidates correlated negatively with the antagonistic activity rendering them biologically implausible. In contrast, the candidate with the mass 363.25 Da correlated positively with the antiestrogenic and antiandrogenic activity consistently throughout all experiments (p<0.05, see example in Figure 2). However, we identified three samples that did not conform with this correlation: samples 7 and 11 as well as 8 and 11 induced potent antagonistic activity but the mass 363.25 was detected in low concentrations in those samples in LTQ-Orbitrap experiments 1 and 2, respectively. In general, the correlation with antiandrogenicity was more pronounced than with the antiestrogenic activity because bottled water inhibited androgen receptor more potently (Figure S3 and S4).

Applying this data analysis strategy to the Orbitrap data obtained in the negative mode resulted in peak lists with an initial number of approximately 27,000–30,000 candidates. However, filtering those lists according to the criteria described above did not return valid candidates. Hence, data from negative ionization are not presented here.

### Structural Elucidation and Identification

Data dependent HR-tandem MS experiments and consecutive HR-MS^3^ studies consistently indicate that the parent ion (m/z of 363.25047) fragments into two daughter ions with a m/z of 251.1251 (ion 1) and 139.00004 (ion 2). The neutral loss from parent to ion 1 and from ion 1 to 2 is m/z of 112.12506 and 112.12537, respectively. This corresponds to the loss of two C_8_H_16_ groups (Figure S5).

We then conducted a database search on ChemSpider to identify plausible chemical structures matching the exact mass of the parent ion. We downloaded all structures corresponding to the possible adducts ([M+H]^+^, [M+K]^+^, [M+Na]^+^, [M+NH_4_]^+^). Using Mass Frontier for *in silico* fragmentation we were able to compare the predicted and experimentally observed fragmentation patterns. Defining a mass defect of 0.005 Da as cut-off, eight out of 483 unique chemical structures were predicted to produce fragments matching the ones observed in the MS^n^ experiments (Table S3, S4). These eight compounds are sodium adducts ([M+Na]^+^) with a corrected monoisotopic mass of 340.26136 and an empirical formula of C_20_H_36_O_4_ (mass defect -0.00066 Da). All chemicals are isomers of (2Z)- or (2E)-but-2-enedioate that differ in the structure of their two octyl side chains (Table S4).

### Confirmation

**Figure 3:**
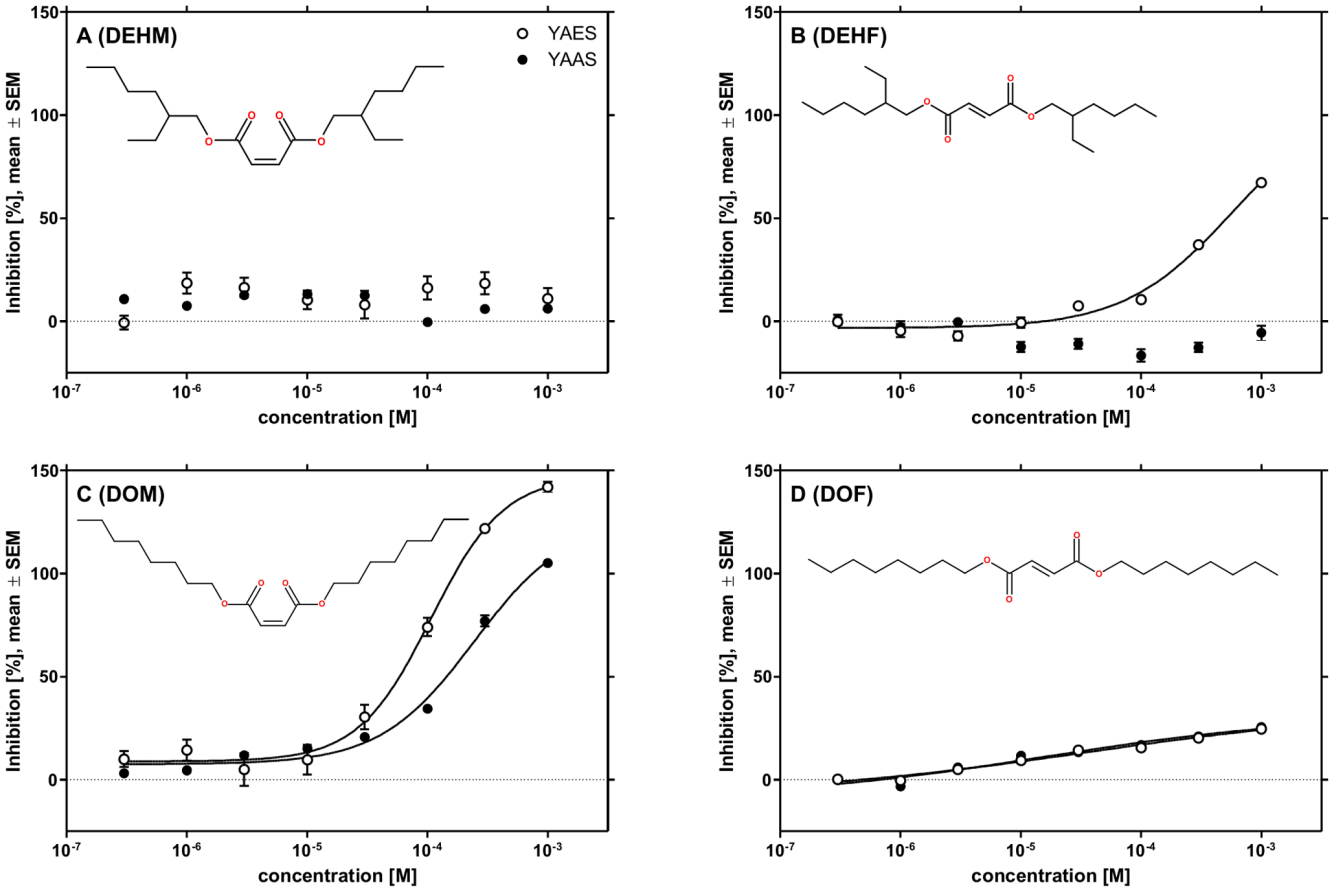
Antiestrogenic and antiandrogenic activity of DEHM (A), DEHF (B), DOM (C), and DOF (D). Data from three experiments with eight replicates each. Dose-response relationships were generated using a four-parameter logistic function.

All isomers of but-2-enedioate possessing two octyl side chains can be expected to conform to the fragmentation pattern observed in the samples. Therefore, we focused on the most common isomers (according to data availability in ChemSpider) and analyzed authentic standards of the maleates DEHM and DOM as well as the fumarates DEHF and DOF using the bioassays and LC-tandem MS. The *in vitro* analysis confirmed that DEHF (IC_50_ = 5.70×10^−4^ M), DOM (IC_50_ = 1.07×10^−4^ M), and DOF (IC_50_ = 3.54×10^−5^ M) are antiestrogenic in the YAES (Figure 3). Moreover, DOM and DOF were antiandrogenic in the YAAS (IC_50_ = 2.45×10^−4^ and 1.58×10^−5^ M, respectively). DEHM was inactive in both bioassays (Figure 3 A).

In the LC-tandem MS (QqQ) analysis, all standards and reanalyzed samples produced concordant SRMs (parent m/z 363.3, ion 1 m/z 251.2, ion 2 m/z 139.0) that were also in accordance with the theoretical prediction (see Figure S6, S7). Retention times (RT) were 27.78, 27.83, and 28.33 min for DEHM, DEHF, and DOM, respectively. DOF was not analyzed. Based on its structure, a longer retention compared to the other isomers is most likely. The samples’ RT (27.88 min) best agrees with the RT of DEHF (see Figure S6). Spiking a bottled water sample with DEHF standard resulted in increased peaks at both SRMs (data not shown). Therefore, chemical analysis confirms with high probability that the molecule with the exact mass of m/z 363.25047 [M+Na]^+^ is DEHF.

## Discussion

### Steroid Receptor Antagonists in Bottled Water

An increasing number of *in vitro* studies reports the presence of EDCs in bottled water [12], [13], [14], [15], [17]. With previous studies focusing on estrogenicity, the present work provides evidence for an additional contamination with steroid receptor antagonists. Using an optimized extraction procedure, we detected antiestrogens and antiandrogens in the majority of analyzed bottled water products. Moreover, the antagonist activity was very potent. An equivalent of 3.75 mL bottled water inhibited estrogen and androgen receptor by up to 60 and 90%, respectively. By deriving bio-equivalents [26], this inhibition can be set in relation to the pharmaceutical antiandrogen flutamide that was used as reference compound in the YAAS (Fig. S8). For the most active samples, the inhibition corresponds to a theoretical concentration of 5.25 mg flutamide equivalents L^−1^. In concordance with our findings, Plotan et al. [16] recently reported antiandrogenicity in one third of the analyzed bottled waters. Here, samples inhibited androgen receptor by approximately 15–70% with the highest activity detected in flavored products.

Similar to our previous study [17], an optimization of the extraction procedure was necessary to isolate steroid receptor antagonists from bottled water effectively. This demonstrates that some commonly used sample preparation techniques are either ineffective in extracting EDCs in quest or effective in coextracting estrogens and antiestrogens that mask each other’s effects. Both scenarios produce false-negative results and might explain the inability to detect endocrine activity in bottled water [18].

From a broader perspective, bottled water from six different countries has been found to contain estrogenic [12], [13], [14], [15], [17], antiestrogenic, and antiandrogenic (this study), as well as androgenic, progestagenic, and glucocorticoid-like chemicals [16]. This demonstrates that a popular beverage is contaminated with diverse-acting EDCs. However, none of the causative chemicals has been identified to date, hindering an evaluation of the toxicological relevance of these findings.

### Combining Nontarget Chemical Analysis and Bioassay Data

To identify the chemical entity causing the antagonistic activity of bottled water we combined analytical and biological data in a novel approach. Traditionally, the effect-directed identification of bioactive chemicals involves time-consuming fractionation of the samples, identification of the active fraction(s) followed by targeted, low-resolution mass spectrometry. Here, we instead processed high-resolution mass spectrometry data to generate peak lists containing all compounds detected in bottled water. We assumed that the peak area of the putative receptor antagonist must be correlated with the biological activity. This assumption is only valid if one compound present in all samples is the major driver of antagonistic activity. In our case, this is tentatively supported by the excellent correlation of antiestrogenic and antiandrogenic effects in all samples. Moreover, the latter clearly implies that the same compound antagonizes both steroid receptors.

Since approximately 1,000 of 25,000 peaks correlated with the antagonistic activity, we applied consistency and plausibility criteria to narrowed this list down to one final candidate. In that step-wise procedure, the mandatory presence of a peak in two extracts per sample and the reexamination of the peak shape proved to be useful. In the end, a molecule with the monosisotopic mass of m/z 363.25047 was consistently correlated with the biological activity rendering it a promising candidate. A search in the ChemSpider database returned 607 entries corresponding to that mass. Hence, we fragmented these compounds *in silico* and compared theoretical and experimentally observed fragmentation patterns. Only sodium adducts of C_20_H_36_O_4_ with two octyl chains (C_6_H_6_) produced concordant fragments. Therefore, we conclude that the final candidate is an isomer of dioctyl maleate or fumarate.

### Identification and Confirmation

Using authentic standards of four common maleates and fumarates we used chemical and biological analysis to confirm the identity of the putative steroid receptor antagonist. LC-tandem MS analysis confirmed with high probability that di(ethylhexyl) fumarate (DEHF) is the isomer in quest. However, in the bioassays DEHF was a weak antagonist of estrogen receptor, only. Albeit the DEHF concentrations are correlated with the samples’ antiestrogenicity, they are far too low (∼250 ng L^−1^) to explain the observed activity. Moreover, DEHF is inactive at androgen receptor. Therefore, chemicals other than DEHF must contribute to the antiestrogenicity and cause the antiandrogenicity we detected in bottled water.

Because DEHF does not ultimately explain the observed antagonistic activity, we need to critically review the limitations of our analysis strategy. While the software-assisted generation of nontargeted peak lists and *in silico* fragmentation proved suitable, the core assumption of the correlation approach is only valid under certain conditions. A compound will correlate with the biological activity if it (a) is present in the majority of samples and (b) is the only or at least the most superior driver of biological activity. DEHF fulfills criterion (a) but misses (b) because of its low antiestrogenicity and lacking antiandrogenicity. From that we can deduce the following:

- The final candidate caused the activity but was misclassified as DEHF. This means another isomer with the same exact mass and retention time – probably also a maleate or fumarate – is the active compound. Because of the multitude of isomers (at least 15,842 isomers are theoretically possible) this option is difficult to verify experimentally.
- DEHF is correlated because it is the detectable proxy of undetected, active compounds. This might be the case if DEHF is part of a contaminant mixture introduced by the same source (e.g., via the surfactant DEHSS, see below). If other active components are present in that mixture but remain undetected, DEHF would be nothing but a statistical representation of these.
- We observed a pseudocorrelation of DEHF and the antagonistic activity. This implies that another undetected compound caused the activity. Alternatively, we might also be dealing with a mixture of steroid receptor antagonists. These chemicals can be readily detected but will not correlate with the activity because they are likely present in different mixture ratios. The latter would also be the case if each sample contains different antagonistic chemicals.

These issues cannot be resolved by data analysis alone but demand alternative experimental approaches: The problem of undetected chemicals requires the use of additional ionization techniques during Orbitrap analysis (e.g., other ion sources). The case of pseudocorrelation can only be explored by a physical fractionation of the samples to deconvolute the effects of the individual mixture constituents. However, although fractionation reduces the number of analytes, it still yields fractions containing numerous chemicals. For instance, when we fine-fractionated leachates of polycarbonate bottles and analyzed the estrogenic fractions in the Orbitrap, we still detected approximately 14,000 candidates (unpublished data). This illustrates that fractionation alone does not resolve the problem. Thus, a combination of physical fractionation and correlation-based data analysis appears adequate to isolate EDCs from complex samples.

### Maleates and Fumarates as Novel Group of Steroid Receptor Antagonists

Keeping the limitations of this study in mind, it is, however, possible that a C_20_H_36_O_4_ isomer other than DEHF is the causative steroid receptor antagonist in bottled water. In principle, every molecule with two octyl chains and a C_4_H_2_O_4_ center is a potential candidate, including all, so far untested isomers of dioctyl maleate and fumarate. In the bioassays, we found that unbranched maleates/fumarates antagonized estrogen and androgen receptor, albeit at high concentrations (DOM) or partially (DOF), only. This provides first evidence for the assumption that dialkyl maleates/fumarates might represent a novel group of steroid receptor antagonists. However, future studies are needed to investigate the potential toxicity of this chemical class more thoroughly. These should include bioassays based on mammalian cells to support our findings from yeast-based systems and account for potential toxicokinetic and toxicodynamic differences.

Despite the dearth of published toxicological data, the structural analogy of maleates and phthalates is striking (Fig. S9). Phthalates are widely used plasticizers and well-documented steroid receptor antagonists *in vitro* [23] and antiandrogens *in vivo* [27]. Likewise the fumarates share a certain similarity with adipates (e.g., in the octyl sidechains). Although far less well characterized with regard to endocrine disrupting effects, adipates are promoted as substitute to phthalates. Possessing a chemical structure similar to well-known EDCs, dialkyl maleates and fumarates merit further toxicological evaluation.

### Sources of Maleates and Fumarates

Not much is known about the uses of dialkyl maleates and fumarates. Hence, we can only speculate on its origin in bottled water. DEHM, DEHF, and DOM have been proposed as alternative plasticizers [28], [29], [30] but the actual breadth of application is unclear. Besides polymers, there are other potential sources: Fiselier et al. [31] detected DEHM in µg–mg kg^−1^ amounts in foodstuff (rice, couscous, noodles). Here, the maleate migrated from the cardboard packaging and was found to be an impurity of di(2-ethylhexyl) sulfosuccinate (DEHSS), an emulsifier used in water-based varnishes. DEHSS is not only used in packaging coatings but serves as anionic surfactant in other industrial applications. For instance, it is a component of dispersants used during the Deep Water Horizon oil spill [32] and an authorized wetting agent in beverages and food in the US [33]. In our study, we did not detect DEHSS in bottled water (data not shown). However, since maleates and fumarates are potential impurities and degradation products of DEHSS (Fiselier et al. 2010), the latter may be the original source of DEHF in bottled water.

## Conclusion

We have shown that antiestrogens and antiandrogens are present in the majority of bottled water products. To identify the causative chemical, we applied a novel correlation approach to integrate biological and high-resolution mass spectrometry data. Structural elucidation led to dioctyl maleate/fumarate isomers as promising candidates. While chemical analysis confirmed that DEHF is the putative steroid receptor antagonist, this compound was weakly antiestrogenic in the bioassays, only. We conclude that we have either missed active compound(s) or that another; untested maleate/fumarate isomer causes the antagonistic activity in bottled water. Two arguments support the latter: In addition to DEHF other isomers were antiestrogenic and antiandrogenic. Moreover, maleates are structurally highly similar to phthalate plasticizers, well-known antiandrogens. Therefore, we pose the hypothesis that dialkyl maleates and fumarates might represent a novel group of steroid receptor antagonists. This illustrates that in spite of the potentially relevant exposure and obvious resemblance to other EDCs such chemicals have been so far disregarded by the scientific and regulatory community. Therefore, we hope that our findings will give fresh impetus to the effect-directed identification of EDCs in beverages, foodstuff, and consumer products which, in the end, will help providing a more holistic picture of human exposure to EDCs.

## Supporting Information

Figure S1 **Antiestrogenic activity of the materials used for the solid phase extraction in the YAES.** The solvents DMSO, acetone, and methanol (MeOH) and extracts of empty cartridges (C18, Carb, ENV+, HLB, SDB^1^, SDB^XC^) did not induce any significant antiestrogenicity. The antagonistic activity was normalized to controls containing 17β-estradiol (C+E2, 0% inhibition) and such without (C-E2, 100% inhibition).
(TIF)

Figure S2 **Antiestrogenic activity of tap and bottled water (sample 18) extracted with different SPE sorbents (C18, Carb, ENV+, HLB, SDB^1^, SDB^XC^).** The antagonistic activity was normalized to controls containing 17β-estradiol (C+E2, 0% inhibition) and such without (C-E2, 100% inhibition). In the SPE of samples with neutral pH (A) only the ENV+ sorbent was able to extract significant antiestrogenic activity from bottled water (★p<0.05, compared to C+E2). Adjusting the pH of the samples to 2 did not yield antiestrogenic extracts (B).
(TIF)

Figure S3 **Correlation of the antiestrogenic and antiandrogenic activity of bottled water.**
(TIF)

Figure S4 **Correlation of the peak areas (Z-transform) of the three final candidates (m/z 229.14103, 352.09008, and 363.25047) with the antagonistic activity in the YAES and YAAS.** Data sets from the sample extracts analyzed in Orbitrap experiment 1 and 2 are shown here individually. Triangles indicate outliers, the linear regression (with 95% confidence bands) is shown in grey.
(TIF)

Figure S5 **MS^2^ (A) and MS^3^ (B) fragmentation pattern of the molecule with the exact mass of 363.25047 (in sample 18).**
(TIF)

Figure S6 **Comparison of retention times and MRMs of a sample and authentic standards.**
(TIF)

Figure S7 **Proposed fragmentation mechanism of but-2-enedioate isomers, illustrated by the example of DOM.**
(TIF)

Figure S8 **Dose-response relationships of hydroxytamoxifen (A) and flutamide (B) used as reference compounds in the YAES and YAAS, respectively.** 95% confidence bands are shown in grey.
(TIFF)

Figure S9 **Structures of maleates (DOM, DEHM) and fumarates (DOF, DEHF) compared to phthalates (di-n-octyl phthalate, DOP; di(2-ethylhexyl) phthalate, DEHP) and adipates (di-n-octyl adipate, DOA; di(2-ethylhexyl) adipate, DEHA), respectively.**
(TIF)

Table S1 **Strategy for processing, combining, and filtering the analytical and biological data to identify candidates causing the antagonistic activity in bottled water.**
(DOCX)

Table S2 **67 candidates detected in both Orbitrap experiments correlated significantly with the antiestrogenic and/or antiandrogenic activity in the YAES and YAAS.** Additionally, the evaluation of each candidate in the following filtering procedure (plausibility of correlation, XIC and scatter plots) is shown.
(DOCX)

Table S3 **Database hits for different adducts of the exact mass of 363.25047.**
(DOCX)

Table S4 **Compounds with an exact mass of 363.25047 [M+Na]^+^ and consistent** ***in silico*** **and experimental fragmentation.**
(DOCX)

Table S5 **Parameters for confirmation studies via LC-tandem MS.**
(DOCX)

Text S1 **Supporting text.**
(DOCX)
