# Supplementary figures and images for: Identification of Putative Steroid Receptor Antagonists in Bottled Water: Combining Bioassays and High-Resolution Mass Spectrometry
Source: PLoS One. 2013 Aug 28;8(8):e72472. doi: 10.1371/journal.pone.0072472 (PMC3756062; doi:10.1371/journal.pone.0072472)

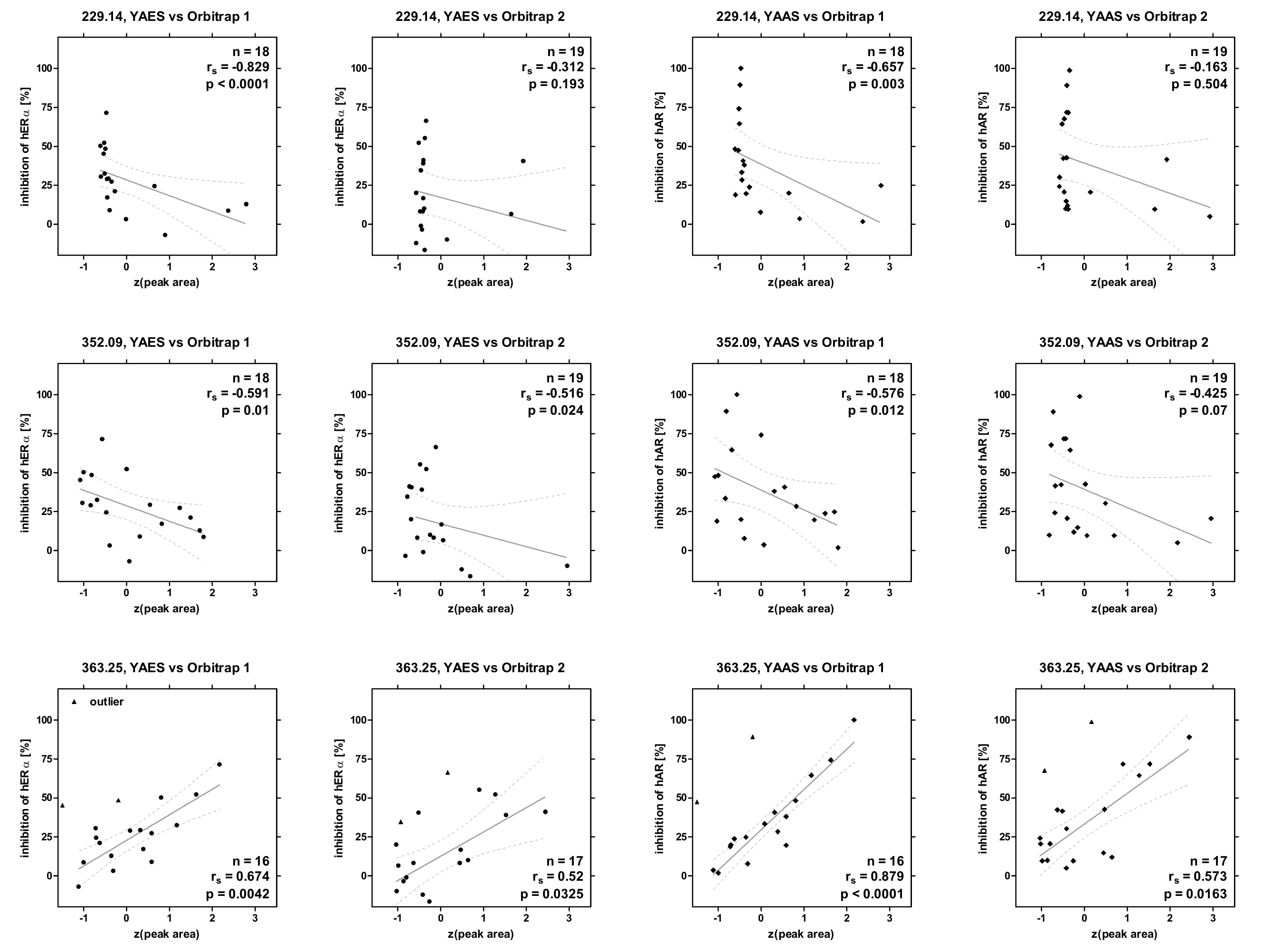

Supplement: Figure S4 — Correlation of the peak areas (Z-transform) of the three final candidates (m/z 229.14103, 352.09008, and 363.25047) with the antagonistic activity in the YAES and YAAS. Data sets from the sample extracts analyzed in Orbitrap experiment 1 and 2 are shown here individually. Triangles indicate outliers, the linear regression (with 95% confidence bands) is shown in grey. (TIF) [file pone.0072472.s004.tif]

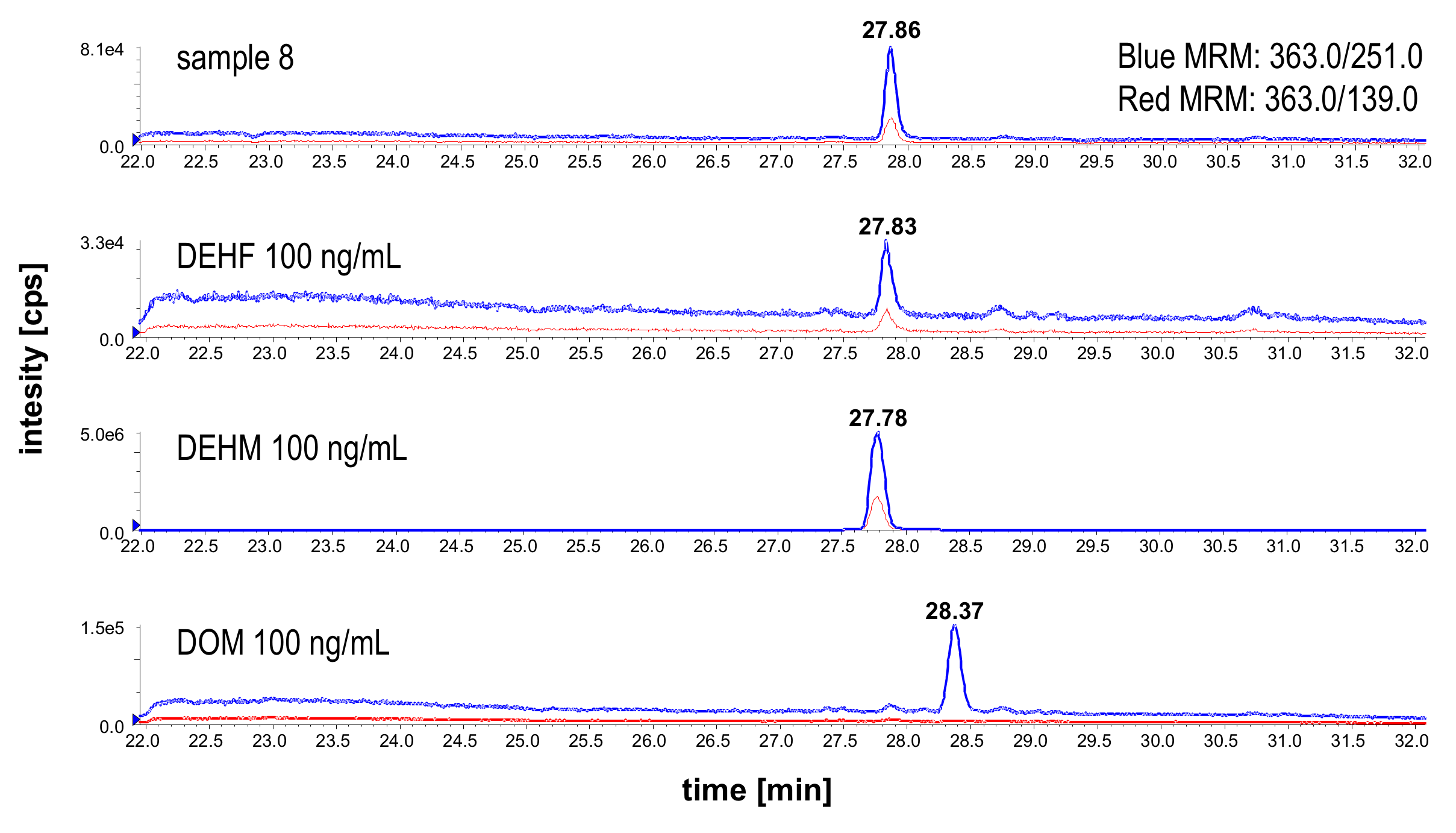

Supplement: Figure S6 — Comparison of retention times and MRMs of a sample and authentic standards. (TIF) [file pone.0072472.s006.tif]

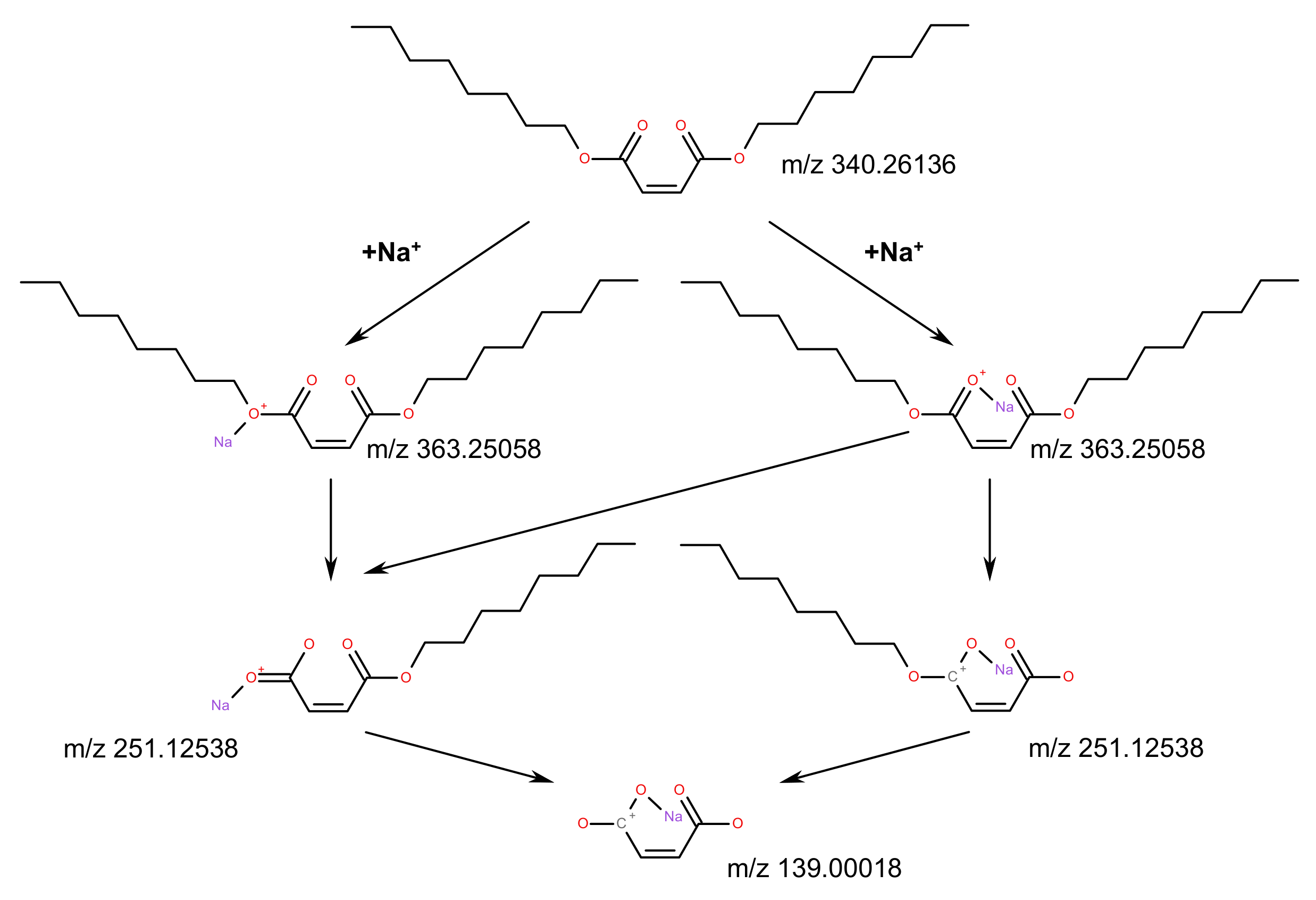

Supplement: Figure S7 — Proposed fragmentation mechanism of but-2-enedioate isomers, illustrated by the example of DOM. (TIF) [file pone.0072472.s007.tif]

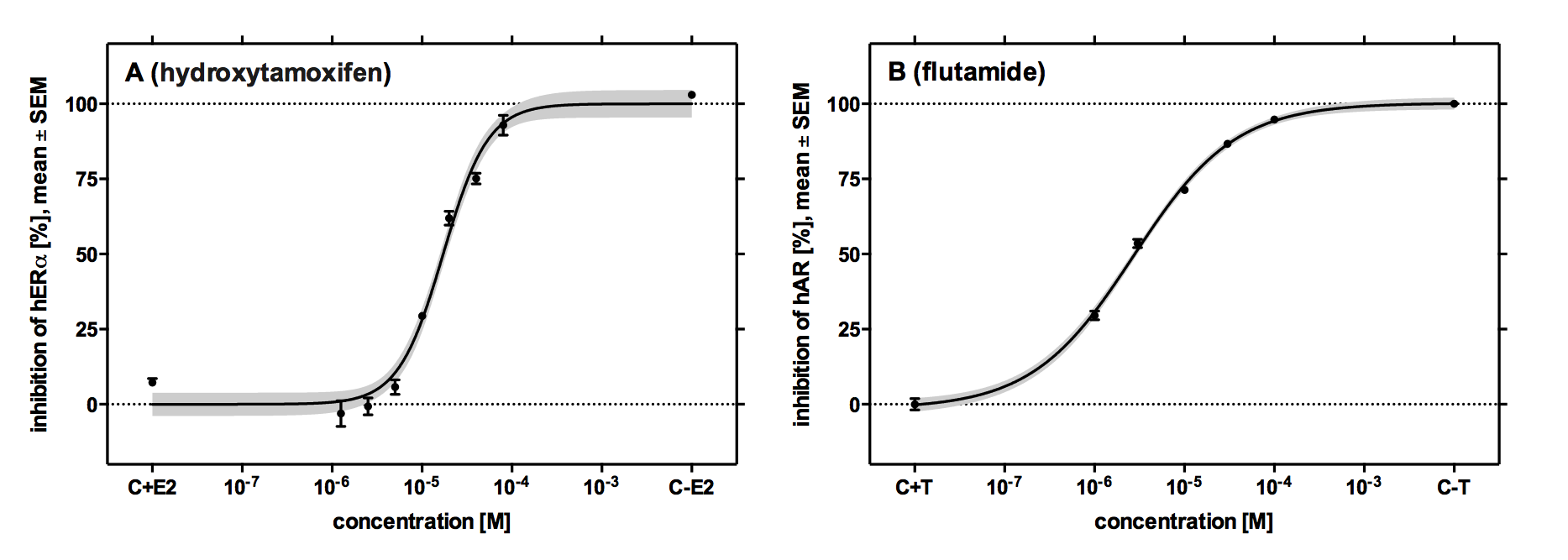

Supplement: Figure S8 — Dose-response relationships of hydroxytamoxifen (A) and flutamide (B) used as reference compounds in the YAES and YAAS, respectively. 95% confidence bands are shown in grey. (TIFF) [file pone.0072472.s008.tif]

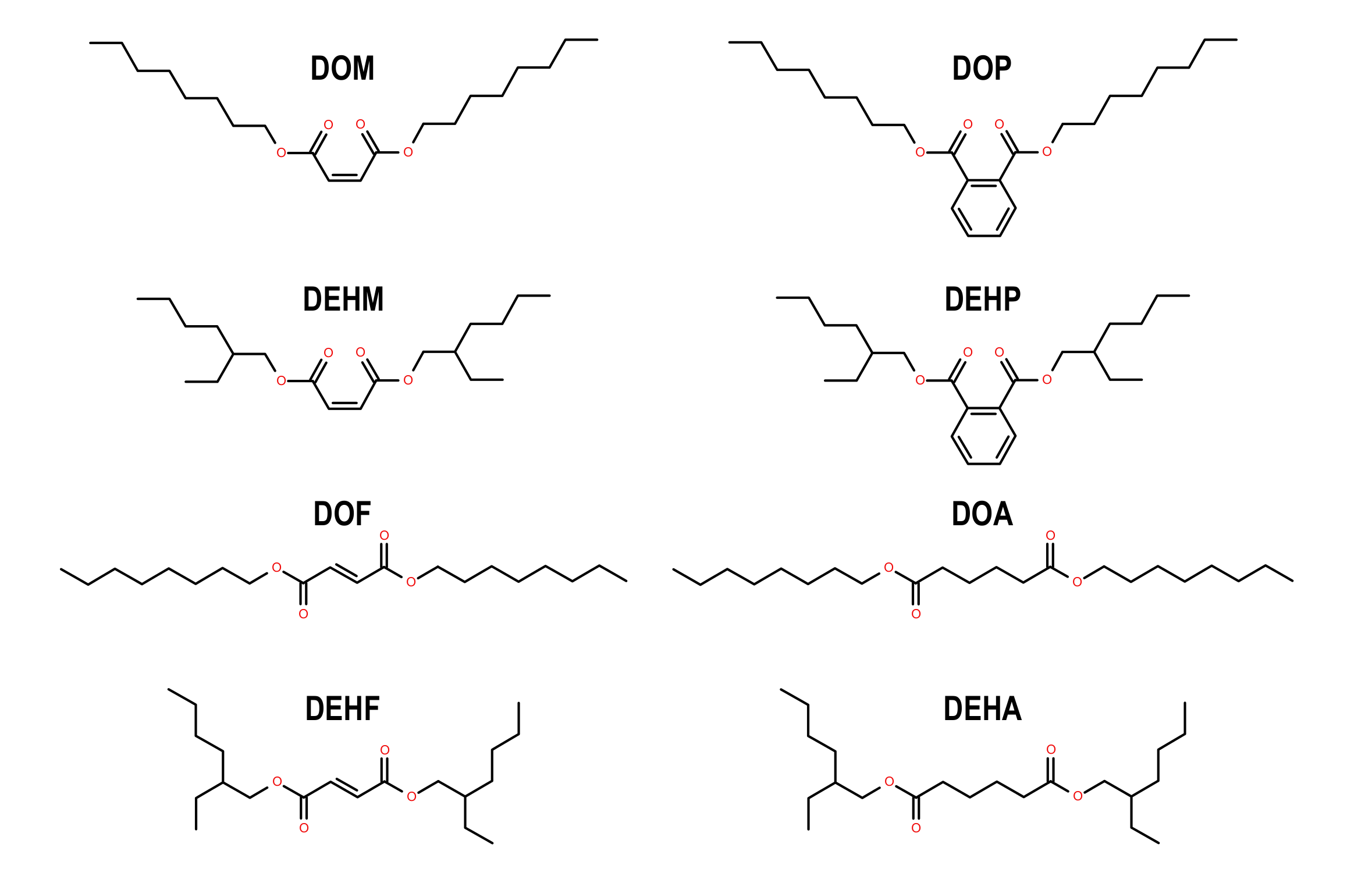

Supplement: Figure S9 — Structures of maleates (DOM, DEHM) and fumarates (DOF, DEHF) compared to phthalates (di-n-octyl phthalate, DOP; di(2-ethylhexyl) phthalate, DEHP) and adipates (di-n-octyl adipate, DOA; di(2-ethylhexyl) adipate, DEHA), respectively. (TIF) [file pone.0072472.s009.tif]
