# Supplementary material for: Identification of Putative Steroid Receptor Antagonists in Bottled Water: Combining Bioassays and High-Resolution Mass Spectrometry
Source: PLoS One. 2013 Aug 28;8(8):e72472. doi: 10.1371/journal.pone.0072472 (PMC3756062; doi:10.1371/journal.pone.0072472)
Supplement: Table S1 — Strategy for processing, combining, and filtering the analytical and biological data to identify candidates causing the antagonistic activity in bottled water. (DOCX) [file pone.0072472.s010.docx]

**Table S1.** Strategy for processing, combining, and filtering the analytical and biological data to identify candidates causing the antagonistic activity in bottled water.

| **data processing step** | **rationale** | **no. of candidates** |
| --- | --- | --- |
| generate peak lists from two Orbitrap data sets using MZmine | retrieve peak information from analytical raw data in an automated/standardized manner | 15593 and 24520 |
| filter for peaks present in at least 12 samples | peaks present in less that 60% of the samples are implausible | 12466 and 18685 |
| correlation of individual peak areas with bioassay data (inhibition in the YAES and YAAS) | peak area (i.e. concentration) of the candidate must be correlated with the antagonistic activity in the samples | 938 and 1066 |
| filter for peaks present in both Orbitrap experiments | peaks detected in one of two extracts only are rejected as candidates | 67 (Table S2) |
| filter for peaks with consistent correlation in both Orbitrap experiments | peaks with conflicting correlation (positive and negative r) in the two extracts are implausible | 43 |
| filter for peaks with plausible XIC | exclude peaks that MZmine generated from noise in the chromatograms | 3 |
| reanalyze peak area, retention time, and m/z from raw data | accounts for potential variances in the automatic peak detection | 3 (Figure S4) |
| assess scatter plots of remaining candidates | final check of the correlations’ plausibility and goodness | 1 (Figure 2, S4) |
