# Supplementary material for: Identification of Putative Steroid Receptor Antagonists in Bottled Water: Combining Bioassays and High-Resolution Mass Spectrometry
Source: PLoS One. 2013 Aug 28;8(8):e72472. doi: 10.1371/journal.pone.0072472 (PMC3756062; doi:10.1371/journal.pone.0072472)
Supplement: Table S2 — 67 candidates detected in both Orbitrap experiments correlated significantly with the antiestrogenic and/or antiandrogenic activity in the YAES and YAAS. Additionally, the evaluation of each candidate in the following filtering procedure (plausibility of correlation, XIC and scatter plots) is shown. (DOCX) [file pone.0072472.s011.docx]

**Table S2.** 67 candidates detected in both Orbitrap experiments correlated significantly with the antiestrogenic and/or antiandrogenic activity in the YAES and YAAS. Additionally, the evaluation of each candidate in the following filtering procedure (plausibility of correlation, XIC and scatter plots) is shown.

| **Orbitrap experiment 1** | | | | | | | |  | **Orbitrap experiment 2** | | | | | | | |  |  |  | |  |
| --- | --- | --- | --- | --- | --- | --- | --- | --- | --- | --- | --- | --- | --- | --- | --- | --- | --- | --- | --- | --- | --- |
|  | | **peak area vs YAES** | | | **peak area vs YAAS** | | |  |  | | **peak area vs YAES** | | | **peak area vs YAAS** | | | delta ppm | correlation consistent | XIC plausible | | scatter plots  plausible |
| mass [m/z] | RT [min] | r | n | p | r | n | p |  | mass [m/z] | RT [min] | r | n | p | r | n | p |  |  | Orbitrap 1 | Orbitrap 2 |  |
| 121.50087 | 6.28 |  |  |  | -0.562 | 13 | 0.046 |  | 121.50130 | 3.49 | -0.549 | 16 | 0.028 |  |  |  | 3.55 | Y | N |  |  |
| 122.49893 | 9.76 | 0.537 | 14 | 0.048 | 0.562 | 14 | 0.037 |  | 122.49960 | 3.30 | -0.529 | 16 | 0.035 |  |  |  | 5.45 | N |  |  |  |
| 133.02511 | 1.49 | 0.622 | 13 | 0.023 |  |  |  |  | 133.02634 | 11.49 |  |  |  | 0.468 | 19 | 0.043 | 9.21 | Y | N |  |  |
| 144.48884 | 13.35 | -0.521 | 15 | 0.046 |  |  |  |  | 144.48893 | 31.95 | 0.527 | 19 | 0.02 | 0.569 | 19 | 0.011 | 0.62 | N |  |  |  |
| 144.98837 | 13.92 | -0.519 | 16 | 0.039 |  |  |  |  | 144.98799 | 5.55 | 0.477 | 18 | 0.046 | 0.527 | 18 | 0.025 | -2.60 | N |  |  |  |
| 156.45447 | 5.78 |  |  |  | 0.684 | 11 | 0.020 |  | 156.45350 | 34.85 |  |  |  | 0.484 | 19 | 0.036 | -6.24 | Y | N |  |  |
| 158.02893 | 1.04 | 0.579 | 13 | 0.038 |  |  |  |  | 158.02888 | 4.65 |  |  |  | 0.600 | 16 | 0.014 | -0.29 | Y | N |  |  |
| 162.97394 | 29.66 | 0.549 | 15 | 0.034 | 0.549 | 15 | 0.034 |  | 162.97396 | 10.79 | -0.547 | 19 | 0.015 |  |  |  | 0.09 | N |  |  |  |
| 162.97409 | 28.92 | 0.572 | 15 | 0.026 |  |  |  |  | 162.97397 | 10.85 | -0.521 | 19 | 0.022 |  |  |  | -0.75 | N |  |  |  |
| 162.97433 | 3.03 | -0.529 | 15 | 0.043 |  |  |  |  | *162.97396* | *10.79* | *-0.547* | *19* | *0.015* |  |  |  | -2.21 | Y | Y | N |  |
| *162.97433* | *3.03* | *-0.529* | *15* | *0.043* |  |  |  |  | *162.97397* | *10.85* | *-0.521* | *19* | *0.022* |  |  |  | -2.21 | Y | Y | N |  |
| 166.96850 | 0.34 | 0.584 | 13 | 0.036 | 0.650 | 13 | 0.016 |  | 166.96871 | 11.35 | -0.476 | 19 | 0.039 |  |  |  | 1.25 | N |  |  |  |
| 170.01574 | 10.39 | 0.618 | 13 | 0.024 | 0.589 | 13 | 0.034 |  | 170.01660 | 2.18 | 0.524 | 15 | 0.045 |  |  |  | 5.10 | Y | N |  |  |
| 176.00817 | 28.29 | 0.579 | 15 | 0.024 |  |  |  |  | 176.00823 | 2.29 | 0.534 | 15 | 0.04 |  |  |  | 0.37 | Y | N |  |  |
| 176.51108 | 16.91 | 0.542 | 14 | 0.045 | 0.577 | 14 | 0.031 |  | 176.51117 | 2.32 | 0.533 | 15 | 0.041 |  |  |  | 0.52 | Y | N |  |  |
| 177.00636 | 28.37 | 0.661 | 12 | 0.019 |  |  |  |  | 177.00642 | 4.15 | -0.595 | 16 | 0.015 |  |  |  | 0.38 | N |  |  |  |
| 177.00645 | 13.95 | 0.673 | 13 | 0.012 | 0.716 | 13 | 0.006 |  | *177.00642* | *4.15* | *-0.595* | *16* | *0.015* |  |  |  | -0.13 | N |  |  |  |
| 183.01654 | 7.57 | 0.595 | 14 | 0.025 |  |  |  |  | 183.01672 | 4.64 | 0.550 | 16 | 0.027 | 0.531 | 16 | 0.034 | 1.00 | Y | N |  |  |
| 183.01728 | 9.68 | 0.788 | 13 | 0.001 | 0.743 | 13 | 0.004 |  | *183.01672* | *4.64* | *0.550* | *16* | *0.027* | *0.531* | *16* | *0.034* | -3.04 | Y | N |  |  |
| 189.03541 | 29.96 |  |  |  | -0.626 | 13 | 0.022 |  | 189.03500 | 22.43 |  |  |  | 0.480 | 19 | 0.037 | -2.14 | N |  |  |  |
| 191.02216 | 5.85 | 0.557 | 14 | 0.039 |  |  |  |  | 191.02157 | 4.64 | 0.687 | 16 | 0.003 | 0.545 | 16 | 0.029 | -3.09 | Y | N |  |  |
| 198.18502 | 18.58 | -0.672 | 13 | 0.012 |  |  |  |  | 198.18454 | 13.01 | -0.479 | 19 | 0.038 |  |  |  | -2.44 | Y | N |  |  |
| 199.01004 | 9.57 | -0.608 | 14 | 0.021 |  |  |  |  | 199.01085 | 3.91 | -0.610 | 16 | 0.012 |  |  |  | 4.08 | Y | N |  |  |
| 204.53550 | 13.09 | 0.614 | 15 | 0.015 |  |  |  |  | 204.53571 | 6.92 |  |  |  | 0.475 | 19 | 0.04 | 1.03 | Y | N |  |  |
| 204.53641 | 8.76 | 0.767 | 12 | 0.004 | 0.597 | 12 | 0.040 |  | *204.53571* | *6.92* |  |  |  | *0.475* | *19* | *0.04* | -3.42 | Y | N |  |  |
| 223.09436 | 22.48 |  |  |  | 0.576 | 13 | 0.039 |  | 223.09213 | 34.02 | 0.578 | 19 | 0.01 | 0.539 | 19 | 0.017 | -9.99 | Y | N |  |  |
| 225.00952 | 8.03 | -0.587 | 13 | 0.035 |  |  |  |  | 225.00992 | 5.30 | -0.537 | 18 | 0.022 |  |  |  | 1.77 | Y | N |  |  |
| 229.13974 | 16.96 | -0.499 | 18 | 0.035 | -0.495 | 18 | 0.037 |  | 229.14108 | 14.88 |  |  |  | -0.463 | 19 | 0.046 | 5.89 | Y | Y | Y | N |
| 236.07130 | 28.22 | 0.675 | 14 | 0.008 | 0.607 | 14 | 0.021 |  | 236.07179 | 16.11 | 0.522 | 19 | 0.022 | 0.533 | 19 | 0.019 | 2.07 | Y | N |  |  |
| 237.07705 | 32.98 | -0.572 | 16 | 0.021 | -0.674 | 16 | 0.004 |  | 237.07527 | 33.00 | -0.538 | 19 | 0.017 |  |  |  | -7.53 | Y | Y | N |  |
|  |  |  |  |  |  |  |  |  |  |  |  |  |  |  |  |  |  |  |  |  |  |
| **Table S2 continued.** | | |  |  |  |  |  |  |  |  |  |  |  |  |  |  |  |  |  |  |  |
| **Orbitrap experiment 1** | | | | | | | |  | **Orbitrap experiment 2** | | | | | | | |  |  |  | |  |
|  | | **peak area vs YAES** | | | **peak area vs YAAS** | | |  |  | | **peak area vs YAES** | | | **peak area vs YAAS** | | | delta ppm | correlation consistent | XIC plausible | | scatter plots  plausible |
| mass [m/z] | RT [min] | r | n | p | r | n | p |  | mass [m/z] | RT [min] | r | n | p | r | n | p |  |  | Orbitrap 1 | Orbitrap 2 |  |
| 240.98746 | 13.41 | 0.759 | 14 | 0.002 | 0.536 | 14 | 0.048 |  | 240.98747 | 12.70 | -0.477 | 19 | 0.039 |  |  |  | 0.01 | N |  |  |  |
| 240.98748 | 11.27 | 0.688 | 13 | 0.009 | 0.569 | 13 | 0.042 |  | *240.98747* | *12.70* | *-0.477* | *19* | *0.039* |  |  |  | -0.05 | N |  |  |  |
| 243.13513 | 20.22 | -0.694 | 13 | 0.008 | -0.589 | 13 | 0.034 |  | 243.13559 | 26.76 | -0.518 | 19 | 0.023 |  |  |  | 1.90 | Y | N |  |  |
| 243.54267 | 12.92 | 0.555 | 13 | 0.049 | 0.714 | 13 | 0.006 |  | 243.54277 | 32.11 | 0.556 | 18 | 0.017 |  |  |  | 0.41 | Y | N |  |  |
| 243.54277 | 6.60 | 0.673 | 14 | 0.008 |  |  |  |  | *243.54277* | *32.11* | *0.556* | *18* | *0.017* |  |  |  | 0.00 | Y | N |  |  |
| 244.94602 | 9.51 | 0.589 | 12 | 0.044 |  |  |  |  | 244.94742 | 32.36 |  |  |  | -0.507 | 19 | 0.027 | 5.74 | N |  |  |  |
| 257.97902 | 4.99 | 0.573 | 13 | 0.041 |  |  |  |  | 257.97912 | 9.94 |  |  |  | 0.529 | 19 | 0.02 | 0.36 | Y | N |  |  |
| 263.05575 | 27.04 | 0.644 | 13 | 0.017 | 0.584 | 13 | 0.036 |  | 263.05675 | 22.78 | -0.492 | 19 | 0.033 |  |  |  | 3.82 | N |  |  |  |
| 279.54593 | 29.89 | 0.712 | 14 | 0.004 | 0.669 | 14 | 0.009 |  | 279.54657 | 28.80 | -0.478 | 19 | 0.039 |  |  |  | 2.30 | N |  |  |  |
| 288.55157 | 29.84 |  |  |  | 0.631 | 12 | 0.028 |  | 288.55199 | 28.80 | -0.584 | 16 | 0.018 |  |  |  | 1.43 | N |  |  |  |
| 295.19591 | 30.14 | 0.726 | 13 | 0.005 | 0.703 | 13 | 0.007 |  | 295.19701 | 15.74 | -0.511 | 19 | 0.025 |  |  |  | 3.71 | N |  |  |  |
| 299.18451 | 26.02 | 0.724 | 13 | 0.005 | 0.609 | 13 | 0.027 |  | 299.18495 | 30.05 | 0.466 | 19 | 0.044 | 0.512 | 19 | 0.025 | 1.49 | Y | N |  |  |
| 305.26373 | 31.61 |  |  |  | -0.549 | 15 | 0.034 |  | 305.26510 | 30.01 |  |  |  | 0.498 | 19 | 0.03 | 4.50 | N |  |  |  |
| 313.23451 | 27.93 | 0.832 | 13 | 0.000 | 0.652 | 13 | 0.016 |  | 313.23532 | 25.49 | -0.576 | 19 | 0.01 |  |  |  | 2.59 | N |  |  |  |
| 313.36540 | 24.91 |  |  |  | 0.585 | 13 | 0.036 |  | 313.36591 | 23.92 |  |  |  | 0.487 | 19 | 0.034 | 1.63 | Y | N |  |  |
| 318.05308 | 29.84 | 0.717 | 14 | 0.004 | 0.721 | 14 | 0.004 |  | 318.05340 | 28.80 | -0.560 | 17 | 0.019 |  |  |  | 1.01 | N |  |  |  |
| 318.55237 | 29.90 | 0.676 | 13 | 0.011 | 0.695 | 13 | 0.008 |  | 318.55394 | 28.80 | -0.508 | 16 | 0.045 |  |  |  | 4.93 | N |  |  |  |
| 321.21679 | 31.68 | 0.599 | 13 | 0.031 | 0.567 | 13 | 0.043 |  | 321.21670 | 28.97 | 0.505 | 19 | 0.027 | 0.495 | 19 | 0.031 | -0.27 | Y | N |  |  |
| 329.36054 | 29.31 | -0.549 | 14 | 0.042 |  |  |  |  | 329.36059 | 25.16 |  |  |  | 0.610 | 19 | 0.006 | 0.16 | N |  |  |  |
| 329.36092 | 29.83 | -0.682 | 14 | 0.007 |  |  |  |  |  |  |  |  |  |  |  |  | -1.00 | N |  |  |  |
| 340.32043 | 24.71 | 0.625 | 13 | 0.022 |  |  |  |  | 340.32134 | 27.37 | -0.550 | 19 | 0.015 | -0.498 | 19 | 0.03 | 2.68 | N |  |  |  |
| 343.20851 | 29.07 | 0.611 | 13 | 0.026 |  |  |  |  | 343.20927 | 22.71 |  |  |  | 0.509 | 19 | 0.026 | 2.23 | Y | N |  |  |
| 343.20961 | 25.31 | 0.648 | 13 | 0.017 | 0.594 | 13 | 0.032 |  | *343.20927* | *22.71* |  |  |  | *0.509* | *19* | *0.026* | -0.99 | Y | N |  |  |
| 344.21277 | 24.38 | 0.546 | 16 | 0.029 | 0.560 | 16 | 0.024 |  | 344.21396 | 29.15 |  |  |  | 0.475 | 19 | 0.04 | 3.45 | Y | N |  |  |
| 348.18700 | 22.88 | 0.555 | 14 | 0.040 |  |  |  |  | 348.18728 | 24.21 |  |  |  | 0.597 | 19 | 0.007 | 0.83 | Y | N |  |  |
| 352.09023 | 22.97 |  |  |  | -0.527 | 16 | 0.036 |  | 352.09102 | 20.62 | -0.575 | 19 | 0.01 |  |  |  | 2.23 | Y | Y | Y | N |
| 353.26609 | 24.51 | -0.552 | 14 | 0.041 |  |  |  |  | 353.26634 | 1.77 |  |  |  | -0.691 | 12 | 0.013 | 0.71 | Y | N |  |  |
| 363.24979 | 27.58 | 0.628 | 13 | 0.022 | 0.680 | 13 | 0.011 |  | 363.24657 | 23.62 | 0.549 | 19 | 0.015 | 0.625 | 19 | 0.004 | -8.88 | Y | Y | Y | Y |
| 373.16375 | 17.54 | 0.687 | 13 | 0.010 | 0.560 | 13 | 0.047 |  | 373.16566 | 15.33 | 0.488 | 19 | 0.034 | 0.482 | 19 | 0.037 | 5.12 | Y | N |  |  |
| 376.31785 | 28.31 | -0.584 | 14 | 0.028 |  |  |  |  | 376.32027 | 21.91 |  |  |  | -0.474 | 19 | 0.04 | 6.44 | Y | N |  |  |
| 377.15563 | 14.19 | 0.675 | 12 | 0.016 | 0.642 | 12 | 0.024 |  | 377.15780 | 11.58 | 0.565 | 19 | 0.012 | 0.644 | 19 | 0.003 | 5.75 | Y | N |  |  |
|  |  |  |  |  |  |  |  |  |  |  |  |  |  |  |  |  |  |  |  |  |  |
| **Table S2 continued.** | | |  |  |  |  |  |  |  |  |  |  |  |  |  |  |  |  |  |  |  |
| **Orbitrap experiment 1** | | | | | | | |  | **Orbitrap experiment 2** | | | | | | | |  |  |  | |  |
|  | | **peak area vs YAES** | | | **peak area vs YAAS** | | |  |  | | **peak area vs YAES** | | | **peak area vs YAAS** | | | delta ppm | correlation consistent | XIC plausible | | scatter plots  plausible |
| mass [m/z] | RT [min] | r | n | p | r | n | p |  | mass [m/z] | RT [min] | r | n | p | r | n | p |  |  | Orbitrap 1 | Orbitrap 2 |  |
| 377.15946 | 16.83 | 0.657 | 13 | 0.015 |  |  |  |  | 377.16299 | 13.68 | 0.463 | 19 | 0.046 |  |  |  | 9.35 | Y | N |  |  |
| 377.16316 | 18.62 | 0.596 | 13 | 0.031 |  |  |  |  | *377.16299* | *13.68* | *0.463* | *19* | *0.046* |  |  |  | -0.47 | Y | N |  |  |
| 377.30236 | 27.62 |  |  |  | -0.535 | 14 | 0.049 |  | 377.30429 | 24.36 |  |  |  | 0.525 | 19 | 0.021 | 5.11 | N |  |  |  |
| 385.16153 | 16.09 | 0.556 | 13 | 0.048 |  |  |  |  | 385.16179 | 15.02 | 0.484 | 19 | 0.036 | 0.476 | 19 | 0.039 | 0.67 | Y | N |  |  |
| 385.16436 | 21.01 | 0.606 | 13 | 0.028 |  |  |  |  | 385.16246 | 15.47 |  |  |  | 0.528 | 19 | 0.02 | -4.93 | Y | N |  |  |
| 399.17439 | 17.12 | 0.563 | 13 | 0.045 |  |  |  |  | 399.17373 | 25.12 | 0.472 | 18 | 0.048 |  |  |  | -1.64 | Y | N |  |  |
| 419.16767 | 16.29 | 0.709 | 12 | 0.010 | 0.605 | 12 | 0.037 |  | 419.16803 | 13.95 | 0.498 | 19 | 0.03 |  |  |  | 0.86 | Y | Y |  |  |
| 427.17545 | 19.14 | 0.553 | 14 | 0.040 |  |  |  |  | 427.17604 | 15.26 |  |  |  | 0.506 | 19 | 0.027 | 1.38 | Y | N |  |  |
| 427.17939 | 18.61 | 0.644 | 13 | 0.017 |  |  |  |  | *427.17604* | *15.26* |  |  |  | *0.506* | *19* | *0.027* | -7.85 | Y | Y | N |  |
| 429.18387 | 16.41 |  |  |  | 0.692 | 12 | 0.013 |  | 429.18320 | 15.62 |  |  |  | 0.492 | 19 | 0.032 | -1.56 | Y | N |  |  |
| 441.18668 | 16.85 | 0.587 | 12 | 0.045 |  |  |  |  | 441.18544 | 18.73 | 0.625 | 19 | 0.004 | 0.630 | 19 | 0.004 | -2.79 | Y | N |  |  |
| *441.18668* | *16.85* | *0.587* | *12* | *0.045* |  |  |  |  | 441.19008 | 15.95 |  |  |  | 0.470 | 19 | 0.042 | 7.72 | Y | N |  |  |
| 447.19466 | 16.87 | 0.584 | 13 | 0.036 |  |  |  |  | 447.19540 | 22.62 | -0.480 | 18 | 0.044 |  |  |  | 1.66 | N |  |  |  |
| 447.19780 | 18.56 | 0.641 | 12 | 0.025 |  |  |  |  |  |  |  |  |  |  |  |  | -5.35 | N |  |  |  |
| 467.10105 | 20.68 | 0.570 | 13 | 0.042 |  |  |  |  | 467.10420 | 17.01 | 0.498 | 19 | 0.03 | 0.628 | 19 | 0.004 | 6.75 | Y | N |  |  |
| 507.32820 | 24.54 |  |  |  | 0.671 | 18 | 0.002 |  | 507.32940 | 23.09 |  |  |  | -0.471 | 19 | 0.042 | 2.36 | N |  |  |  |
| 536.16448 | 29.85 | 0.518 | 17 | 0.033 | 0.532 | 17 | 0.028 |  | 536.16713 | 28.80 | -0.560 | 19 | 0.013 |  |  |  | 4.93 | N |  |  |  |
| 679.58403 | 28.35 |  |  |  | -0.633 | 13 | 0.020 |  | 679.58681 | 27.22 | -0.527 | 19 | 0.02 | -0.474 | 19 | 0.04 | 4.09 | Y | N |  |  |
