# Supplementary material for: Identification of Putative Steroid Receptor Antagonists in Bottled Water: Combining Bioassays and High-Resolution Mass Spectrometry
Source: PLoS One. 2013 Aug 28;8(8):e72472. doi: 10.1371/journal.pone.0072472 (PMC3756062; doi:10.1371/journal.pone.0072472)
Supplement: Table S3 — Database hits for different adducts of the exact mass of 363.25047. (DOCX) [file pone.0072472.s012.docx]

**Table S3.** Database hits for different adducts of the exact mass of 363.25047.

| **adduct** | **no. of hits in Chemspider** | **empirical formula of the parent (mass defect)** | **no. of unique chemical structures** | **no. of structures with matching *in silico* and experimental fragmentation** |
| --- | --- | --- | --- | --- |
| [M+H]^+^ | 78 | 1 x C_20_H_35_NaO_4_ (0.0001)  1 x C_20_H_33_F_3_O_2_ (0.0001)  76 x C_18_H_30_N_6_O_2_ (-0.0002) | 69 | 0 |
| [M+K]^+^ | 0 | - | - | - |
| [M+Na]^+^ | 254 | 254 x C_20_H_36_O_4_ (-0.0002) | 166 | 8 |
| [M+NH_4_]^+^ | 275 | 1 x C_18_H_35_KNaO_2_ (0.0000)  274 x C_18_H_27_N_5_O_2_ (-0.0001) | 248 | 0 |
