# Supplementary material for: Identification of Putative Steroid Receptor Antagonists in Bottled Water: Combining Bioassays and High-Resolution Mass Spectrometry
Source: PLoS One. 2013 Aug 28;8(8):e72472. doi: 10.1371/journal.pone.0072472 (PMC3756062; doi:10.1371/journal.pone.0072472)
Supplement: Table S4 — Compounds with an exact mass of 363.25047 [M+Na]+ and consistent in silico and experimental fragmentation. (DOCX) [file pone.0072472.s013.docx]

**Table S4.** Compounds with an exact mass of 363.25047 [M+Na]^+^ and consistent *in silico* and experimental fragmentation.

| **structure** | **ChemSpider ID**  **CAS** | **systematic name and synonyms** |
| --- | --- | --- |
| 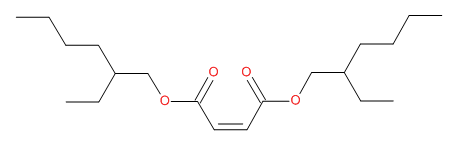 | 4517207  142-16-5 | bis(2-ethylhexyl) (2Z)-but-2-enedioate  di(2-ethylhexyl) maleate  DEHM |
| 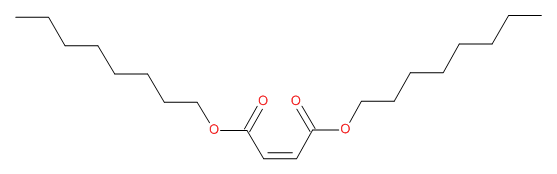 | 4938521  2915-53-9 | dioctyl (2Z)-but-2-enedioate  dioctyl maleate  DOM |
| 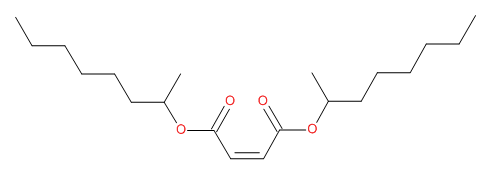 | 4940564  56970-73-1 | bis(1-methylheptyl) (2Z)-but-2-enedioate  bis(1-methylheptyl) maleate |
| 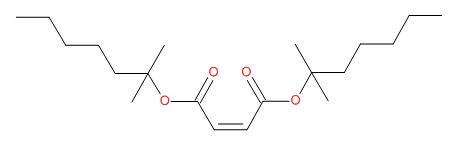 | 5288522  - | bis(1,1-dimethylhexyl) (2Z)-but-2-enedioate  bis(1,1-dimethylhexyl) maleate |
| 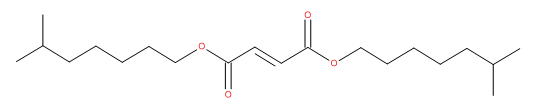 | 4940955  1330-75-2 | bis(6-methylheptyl) (2E)-but-2-enedioate  diisooctyl fumarate |
| 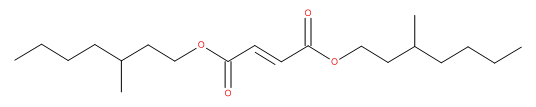 | 192221  - | bis(3-methylheptyl) (2E)-but-2-enedioate |
| 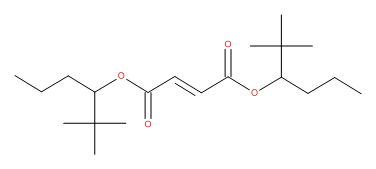 | 14638680  - | bis(1-tert-butylbutyl) (2E)-but-2-enedioate |
| 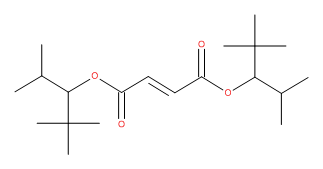 | 14638681  - | bis[2,2-dimethyl-1-(1-methylethyl)propyl] (2E)-but-2-enedioate |
