# Supplementary material for: Identification of Putative Steroid Receptor Antagonists in Bottled Water: Combining Bioassays and High-Resolution Mass Spectrometry
Source: PLoS One. 2013 Aug 28;8(8):e72472. doi: 10.1371/journal.pone.0072472 (PMC3756062; doi:10.1371/journal.pone.0072472)
Supplement: Table S5 — Parameters for confirmation studies via LC-tandem MS. (DOCX) [file pone.0072472.s014.docx]

**Table S5.** Parameters for confirmation studies via LC-tandem MS.

| **Analyte** | **Q1** | **Q3** | **DP [V]** | **CE [eV]** | **CXP [V]** |
| --- | --- | --- | --- | --- | --- |
| DEHF, DOF, DOM, DEHM | 363 363 | 251 139 | 110 110 | 23 28 | 6 6 |
