# Supplementary material for: Identification of Putative Steroid Receptor Antagonists in Bottled Water: Combining Bioassays and High-Resolution Mass Spectrometry
Source: PLoS One. 2013 Aug 28;8(8):e72472. doi: 10.1371/journal.pone.0072472 (PMC3756062; doi:10.1371/journal.pone.0072472)
Supplement: Text S1 — Supporting text. (DOCX) [file pone.0072472.s015.docx]

**Supporting Information for**

**Identification of putative steroid receptor antagonists in bottled water: Combining bioassays and high-resolution mass spectrometry**

Martin Wagner^1^*, Michael P. Schlüsener^2^, Thomas A. Ternes^2^, and Jörg Oehlmann^1^

Chromatographic conditions for Orbitrap and LC-tandem MS studies

Separations were performed using a Luna C18(2) column (2 mm i.d., length 150 mm, particle size 3 µm) and a SecurityGuard (both Phenomenex, Torrance, CA, USA) at 30 ± 2 °C. The flow rate was 0.2 mL/min. The HPLC gradient was established by mixing two mobile phases. Phase A: MilliQ water and phase B: methanol. Chromatographic separation was achieved with the following gradient: 0–1 min: 0% B; 1–19 min: 0🡪100% B; 19–29 min: 100% B; 29–29.1 min: 100🡪0% B; 29.1–35 min: 0% B. 10 µL of each sample was injected.

Confirmation via LC-tandem MS

The tandem MS was operated in positive ion mode using nitrogen as collision gas and multiple reaction monitoring (MRM) for quantification. Parameters adjusted were collision gas (CAD), 6 mTorr; curtain gas (CUR), 20 psi; ion source gas 1 (GS1), 30 psi and ion source gas 2 (GS2), 40 psi; source temperature (TEM), 500 °C; entrance potential (EP), 10 V. The ionspray voltage (IS) was adjusted to 5.5 kV and the interface heater (ihe) set on. Two MRM transitions for each substance were monitored for identification and quantification of the analytes. Parameters such as declustering potential, collision energy, and cell exit potential were optimized in the auto-tuning routine of the Analyst 1.4.2 software.

Table S5 gives an overview of all MS parameters. For chromatographic conditions see above.
